# A Rare Skin Rash Masked by a Bacterial Infection

**DOI:** 10.1177/00099228261444842

**Published:** 2026-04-26

**Authors:** Greta Joskaudaitė, Inga Kisielienė, Raimundas Meškauskas

**Affiliations:** 1 Centre of Dermatovenereology, Vilnius University Hospital Santaros Klinikos, Lithuania; 2 Clinic of Infectious Diseases and Dermatovenereology, Faculty of Medicine, Vilnius University, Lithuania; 3 Children’s Hospital, Affiliate of Vilnius University Hospital Santaros Klinikos, Lithuania; 4 National Center of Pathology, Affiliate of Vilnius University Hospital Santaros Klinikos, Lithuania

**Educational Objectives**

- The diagnosis of eosinophilic pustular folliculitis of infancy (EPFI) can be complicated and delayed due to the wide differential diagnosis and the presence of a secondary infection.
- EPFI is a benign condition that does not require aggressive treatment – the management of exacerbations with topical steroids and antihistamines is effective and safe in pediatric patients.

## Case Report

A 2-year-old boy was presented to the pediatric center because of a pruritic eruption on the scalp that had been recurring since 11 months of age. Previously, repeated microscopic examinations and cultures for fungal and bacterial infections were negative. However, a microbiological culture taken 3 months prior revealed amoxicillin-susceptible *Staphylococcus aureus*. At that time, an infectious etiology was presumed, and amoxicillin therapy was initiated. Unfortunately, the eruptions recurred while the patient was receiving this treatment.

## Discussion

### Hospital Course

**Figure 1.**
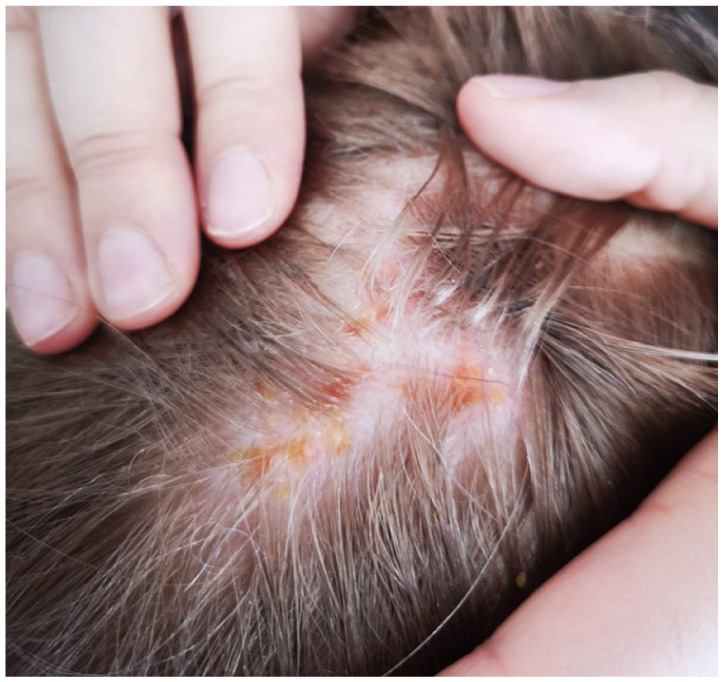
Confluent papules and pustules covered with a yellow crust on the occipital and parietal scalp were observed on admission.

**Figure 2.**
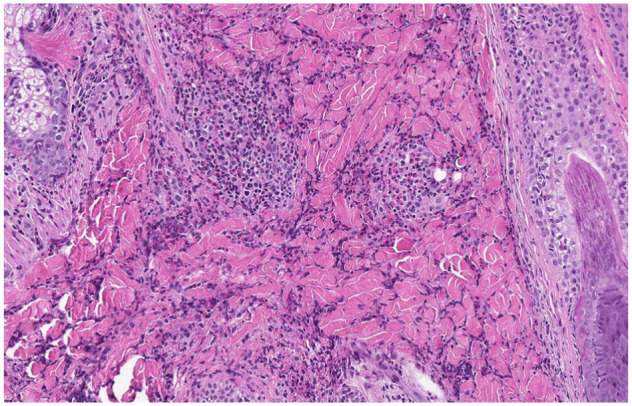
Biopsy showing abundant eosinophilic infiltration in the dermis, where eosinophils spread diffusely between collagen fibers (HE staining, scanned by Aperio, at ×10).

During the physical examination, confluent papules and pustules covered with a yellow crust were observed on the occipital and parietal scalp (Figure 1). After a few weeks, these eruptions spread to the skin of the cheeks and body. During exacerbations, eosinophilia (1.2 × 10^9^/L; range = 0.05-0.70 × 10^9^/L) was detected in the peripheral blood. The patient was consulted by an allergist, but no allergy was found. Due to the atypical clinical presentation of the most common diseases, a biopsy was performed on suspicion of eosinophilic folliculitis. Histopathological examination (Figure 2) revealed epidermal hyperplasia, parakeratosis, and fibrotic papillary dermis. There was abundant monomorphonuclear and eosinophilic interstitial, perifollicular, focal follicular, and pericrine infiltration in the papillary and reticular dermis and adipose tissue. Degenerated collagen fibers were also observed in the reticular dermis. The final diagnosis of eosinophilic pustular folliculitis of infancy (EPFI) was concluded. The patient received therapy with topical corticosteroids (clobetasol and betamethasone combined with fusidic acid) for the cutaneous eruptions once daily, along with oral antihistamine treatment (cetirizine) twice daily.

A favorable therapeutic response was noted during treatment; however, approximately 1 month after discontinuation of antihistamine therapy, a disease flare occurred within 4 to 5 days. Therefore, the treatment was renewed – this time antihistamines were prescribed for a longer period with a gradually tapering scheme, progressing from once-daily dosing to dosing every 2 days for 2 weeks and subsequently to dosing every 3 days for 1 month. After almost a year and a half, a 4-month remission was achieved without the use of antihistamines. Over nearly 4 years of clinical follow-up, episodes of eruptions continue to recur but become less frequent and respond to treatment with topical steroids and antihistamines without side effects.

### Final Diagnosis

The combination of the anamnesis, clinical picture, peripheral blood, and histological findings led to the final diagnosis of EPFI complicated by a secondary bacterial infection.

### Discussion of Case and Literature

Eosinophilic pustular folliculitis of infancy is a rare and benign inflammatory disease characterized by recurrent, sterile, often pruritic pustules and papules on the scalp and less frequently (up to 65%) on other parts of the body, such as the face, trunk, or extremities.^1^ In most cases, the disease tends to appear in the first 14 months of life and more often affects males than females, with a ratio ranging from 3:1 to 4:1.^1,2^ The disease usually resolves in the third year of life (the latest recovery was observed in the ninth year of life), and the frequency of disease exacerbations varies between 1 and 12 weeks. Each exacerbation can last from 7 to 30 days and can sometimes resolve spontaneously.^1^ Although the exact etiopathogenesis is not known, the connection between EPFI with food allergies and medications has been considered as an etiological factor.^3,4^

Eosinophilic pustular folliculitis of infancy poses difficulties in diagnosis because of the wide differential diagnosis, which often delays effective treatment, and in many cases, misdiagnosis leads to excessive and ineffective treatment with systemic drugs.^5,6^ The differential diagnosis should begin with the exclusion of infectious diseases such as impetigo, folliculitis, *herpes simplex*, *varicella zoster*, or fungal infections. Negative cultures and microscopic examination are required to rule out an infectious etiology.^5^ Dermatoscopic or microscopic examination of the skin can help differentiate it from scabies.^2^ As EPFI falls within the spectrum of eosinophilic dermatoses, identification of peripheral blood eosinophilia provides a useful supportive marker that can contribute to establishing the correct diagnosis. Eosinophilia in the blood is observed in up to 83% of cases during flare-ups.^1^ Other conditions that need to be differentiated from EPFI are acropustulosis of infancy, miliaria pustulosa, and Langerhans cell histiocytosis, and when the disease occurs in the neonatal period, erythema toxicum neonatorum and neonatal pustular melanosis should be ruled out.^1,2,5^ Given the similarity of clinical presentations, particularly at initial onset, a comprehensive diagnostic assessment is achieved through evaluation of the totality of clinical signs, disease course, and histopathological features, thereby helping to avoid misdiagnosis.

Histopathological examination in EPFI reveals an eosinophilic infiltrate in the dermis in all cases, often accompanied by eosinophilic microabscesses. The infiltrate in most cases involves the hair follicle or is located perifollicular, perivascular, or interstitial, sometimes around the sweat glands. The infiltrate itself can also be mixed neutrophilic-eosinophilic.^1,2^

Importantly, EPFI can also be combined with another disease, as occurred in the case of our patient – EPFI was complicated by a secondary bacterial (*Staphylococcus aureus*) infection, which delayed the diagnosis. As a result, the patient was treated with systemic antibiotics, which did not have a positive effect on the symptoms of the disease.

Currently, there are no defined guidelines for the treatment of EPFI, but it has been observed that topical steroids (low- to medium-potency) are effective in up to 90% of cases.^1^ Oral antihistamines, such as cetirizine, cimetidine, and hydroxyzine, are also effective in reducing pruritus and the frequency of relapses.^3,4,7^ As an alternative effective treatment, topical calcineurin inhibitors (tacrolimus) have also been described.^8^ Some isolated refractory cases have been successfully treated with dapsone, erythromycin, indomethacin, cyclosporine, or phototherapy. However, due to side effects, most of these drugs are less safe for use in pediatric patients, as opposed to adults.^2,9,10^

In addition, this 4-year follow-up clinical case allowed us to perform a more accurate assessment of the long-term benefit of the applied treatment on disease symptoms and remission. For this patient, treatment with topical steroids and oral antihistamines had a positive effect on symptoms, but after stopping treatment with cetirizine, as in the cases described in the literature, an exacerbation was observed after a few days. Therefore, repeated treatment with oral antihistamines during flare-ups and, in some cases, with a tapering regimen allowed achieving longer remissions even after completing the course with antihistamines.^3,4,7^

## Conclusion

Eosinophilic pustular folliculitis of infancy is a rare disease of unknown etiology with a wide differential diagnosis, making it difficult to recognize. Effective management of disease relapses in the absence of defined treatment guidelines can become a challenge for physicians. Early diagnosis is essential in order to avoid unnecessary and ineffective antimicrobial therapy. After the diagnosis of EPFI is established, it is essential to select not only an effective but also a safe treatment with minimal side effects. In our 4-year observed case, this approach consisted of topical steroids combined with a tapering antihistamine regimen, which allowed for prolonged remissions even after treatment completion.

## Author Contributions

All authors contributed to writing and revising the manuscript.
